# A keratotic nodule on a cobblestone plaque: A rare case presentation

**DOI:** 10.1002/ski2.308

**Published:** 2023-11-09

**Authors:** Nasim Tootoonchi, Mahshid Sadat Ansari, Kambiz Kamyab

**Affiliations:** ^1^ Department of Dermatology Tehran Univeisrty of Medical Sciences Tehran Iran

## Abstract

The manuscript describes a case of a 68‐year‐old woman with a pruritic nodule and yellow cobblestone plaque on her abdomen. Biopsy results showed trans epidermal illumination of basophilic elastic fibres in the reticular dermis with calcium deposits. The diagnosis was perforating calcific elastosis, an acquired rare condition mostly seen in obese, multiparous, middle‐aged women.

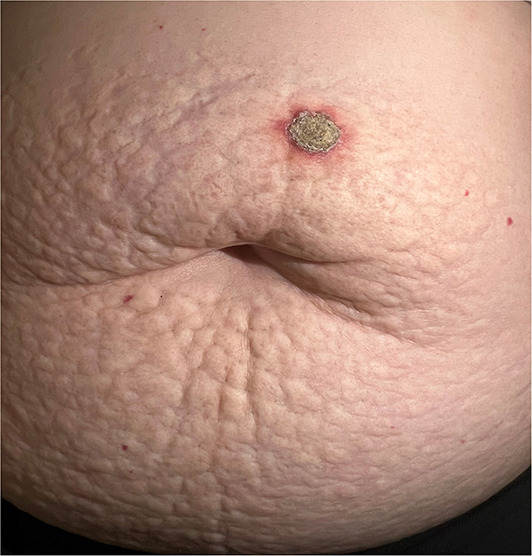

A 68‐year‐old woman presented with near 1‐year pruritic nodule on her abdomen. Examination revealed, a hyperkeratotic erythematous nodule on a background of yellow indurated plaque with cobblestone appearance involving extensive area around umbilicus (Figure 1) Physical exam of other sites was negative for similar lesion. Evaluation of cardiovascular system and eyes was done to exclude pseudoxanthoma elasticum and was unremarkable.

**FIGURE 1:**
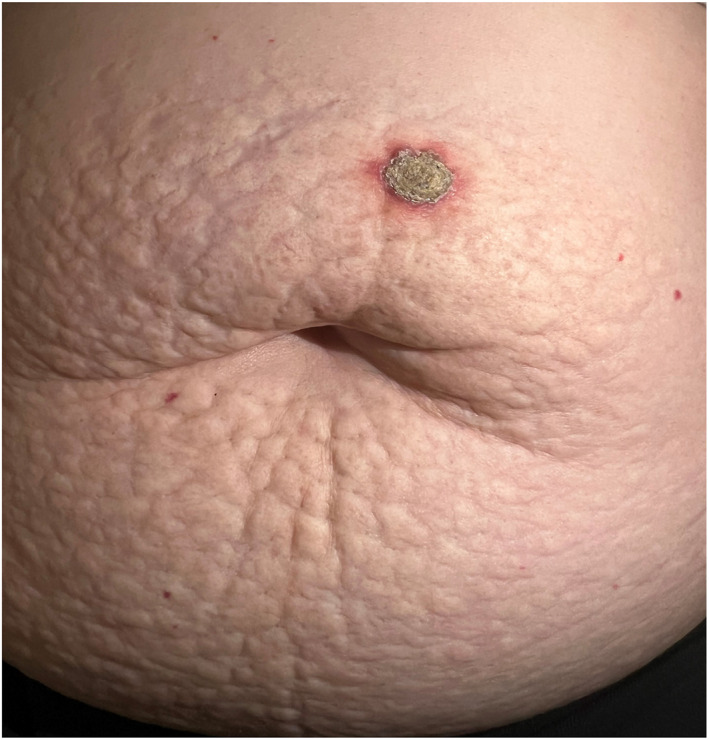
Hyperkeratotic erythematous nodule on a background of yellow indurated plaque with cobblestone appearance in multiparous healthy woman, with no significant past medical history. BMI: 30. (*red papules are cherry angiomas, incidental finding).

Histopathological examination showed curled and basophilic elastic fibres in the reticular dermis. Calcium salts are deposited on the abnormal elastic fibres (Figures 2 and 3). A diagnosis of perforating calcific elastosis was made. Perforating calcific elastosis is an acquired rare condition mostly seen in obese, multiparous, middle‐aged women.^1^

**FIGURE 2:**
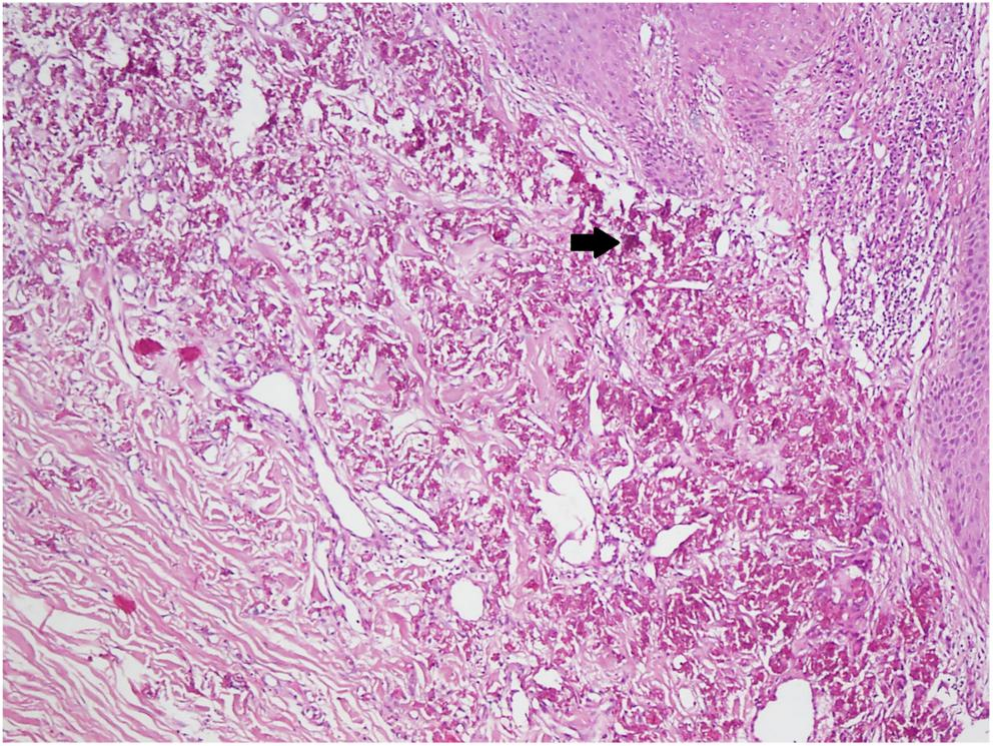
Skin biopsy of both, the keratotic nodule and the white cobblestone plaque was done, stained with Haematoxylin and Eosin (H&E). Histopathological examination showed curled and basophilic elastic fibres (Black Arrow) in the dermis, particularly in the upper and middle parts. Calcium salts are deposited on the abnormal elastic fibres. 40× objective.

**FIGURE 3:**
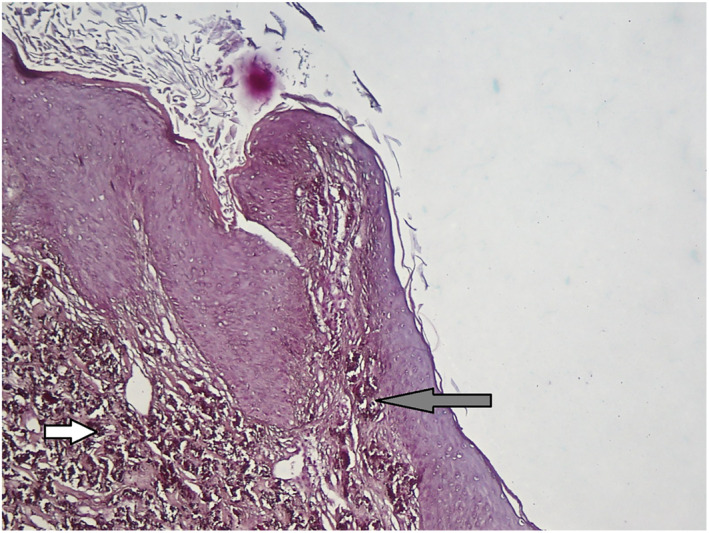
Biopsy of keratotic nodule, revealed abnormal elastic fibres (white arrow) and trans epidermal illumination of elastic fibres (Grey Arrow), stained: Verhoeff‐Van Gieson (VVG). 40× objective.

## CONFLICT OF INTEREST STATEMENT

None to declare.

## AUTHOR CONTRIBUTIONS

**Nasim Tootoonchi**: Conceptualization (lead); investigation (equal). **Mahshid Sadat Ansari**: Formal analysis (equal); writing—original draft (equal); writing—review and editing (lead). **Kambiz Kamyab**: Writing—original draft (equal).

## ETHICS STATEMENT

The patient provided their consent for publication in the journal and the form was obtained.

## Data Availability

The data that support the findings of this study are available from the corresponding author upon reasonable request.
